# On Rheumatism in Its Various Forms, and on the Affections of Internal Organs to Which It Gives Rise

**Published:** 1842-04-01

**Authors:** 


### On Rheumatism in its various Forms, and on the Affections of Internal Organs, more especially the Heart and Brain, to which it gives rise. By *Roderic Macleod,* M.D. Physician to St. George's Hospital. Octavo, pp. 164. Longman and Co. 1842.

The Author of this volume is a plain practical physician, who is not dazzled by, nor dances after, fanciful theories and far-fetched hypotheses; but observes carefully, records faithfully, and reasons closely. A portion of the present small volume formed the substance of the Gulstonian Lectures delivered at the College of Physicians, in 1837 ; but great additions are now incorporated with the original Lectures. The author significantly remarks that "nothing would have been easier than to have doubled the size of the present volume by the insertion of cases ; but the author has been anxious to avoid this *(for reasons which those acquainted with modern medical literature will readily appreciate)*, and has, therefore, restricted himself to a few illustrations required to elucidate some particular subject." —*Pref.*

The numerical returns and calculations are taken exclusively from cases which occurred under the author's care in St. George's Hospital.

The work is divided into fourteen chapters, each of which we shall cursorily notice.

CHAP. I.—Dr. M. considers *atmospheric vicissitudes* as the grand cause of rheumatism, doubting the causation of malaria or other aerial impregnations. The predispositions favouring the operation of these atmospheric transitions, are hereditary tendency—debilitating agents—and former attacks. We may here remark, however, that atmospheric transitions, when accompanied by malarious impregnations, or even simple moisture, are infinitely more operative and injurious than when these are absent. Women are less frequently affected than men, by acute rheumatism, but this seems to result from their less exposure to the causes.

Two thirds of the cases happened between the ages of 15 and 30 years, but no period, from infancy to old age, is exempt from this painful malady. Still it is *comparatively* rare after the age of 50, in its form of rheumatic fever; but in the form of acute, or subacute rheumatic gout, it is very frequent in the advanced epochs of existence.

Its chief seat is the fibrous textures—a wide and extended tissue—sheathing the limbs—constituting the ligaments and tendons—enveloping the brain—and entering into the periosteum. The following are the forms which rheumatism chiefly assumes on external parts :—

"1. The patient may be attacked with pain in one or more joints, with tumefaction and redness, spreading to a greater or less extent over the surrounding parts. The swelling is here external to the joint, the hollows and protuberances about which are obscured, apparently by effusion into the cellular tissue. In this form the disease rapidly shifts its seat, and is accompanied by acute inflammatory fever.

2. In another case the joints likewise are affected, but in a different manner from the preceding. The pain is more limited, and the swelling evidently depends upon effusion into the capsule, which is seen to bulge at those points where the surrounding ligaments present least resistance. The bursæ of the tendons are also frequently implicated, and become distended by an increased effusion of their lubricating fluid. In this form of the disease there is less redness, and usually less violent fever, than in the preceding.

The great practical distinction between these two forms I believe to have been first made by Dr. Chambers : certainly others, who have not always remembered the original source of their information, were, like myself, first taught to make the distinction systematically, when following his practice at St. George's Hospital.

3. In a third form of rheumatism the pain is chiefly referred to parts intermediate between the joints, and seems to be seated in the muscles or their aponeurotic coverings. Here the pain, though it may be exquisite on the slightest movement, is not unfrequently entirely absent when the parts are quiescent: and here, too, there is often little or no constitutional disturbance.

4. In a fourth variety the disease affects the coverings of certain bones, especially those which are but slightly protected by integuments—such as the shin, the ulna, or the cranium: and under such circumstances there are often spots or patches more painful than the rest, tender to the touch, and elevated into nodes.

5. Lastly, the pain sometimes follows the course of particular nerves, more especially those of the lower extremities, and is occasionally confined to a narrow line, which the patient can trace with his finger. In such cases the power of moving the limb is occasionally affected to a greater or less extent.

Now although all these be usually classed together under the general appellation of rheumatism, they are affections so different in their phenomena and treatment, that it is impossible for them to be understood, either theoretically or practically, unless the distinctions alluded to be borne in mind; and without assuming that the textures specified are exclusively affected, or that the names are altogether free from objection, we shall proceed to speak of them as *Rheumatic Fever*, the *Arthritic* or *Capsular*, the *Chronic* or *Muscular*, the *Neuralgic* and the *Periosteal* forms of rheumatism." 11.

### Chap. II.—Rheumatic Fever.

When we consider the great number of cases of rheumatic inflammation which occur in every man's practice, and contemplate the havoc produced by the sequences or consequences of this disease—affections of the head, the heart, pleura, peritoneum, &c.—we may safely say that, next to phthisis, it is the most afflicting scourge of this country. In hospitals, for obvious reasons, these cases accumulate so much that the medical officers of these institutions are apt to consider the disease as still more prevalent than it really is.

In this form pain is rarely absent, but varies in different individuals to a remarkable extent. It is aggravated by motion or pressure—and is always worse in the night, when the sufferer has most need of repose. There are generally pyrexial symptoms, with wandering pains for some hours, or even days, before the localization of the inflammation takes place—when one or more joints become painful, swelled, hot, and red, with remarkable tendency to shift its place from one joint to another. Generally, indeed, the inflammation subsides in a few days from the joint first affected, and springs up in another—thus invading new parts, or returning to its pristine seat. In a small proportion of cases the shoulders, loins, and back are invaded; but seldom present the redness and swelling as in the joints. Very little permanent change remains in the parts after this form of acute rheumatism has subsided—marking its distinction from the synovial, capsular, or arthritic inflammation. The scalp is more frequently the seat of this phlogosis than the integuments of the trunk. Rheumatism of the intercostal spaces and muscles is sometimes mistaken for pleurisy, by young practitioners; but the diagnosis is not difficult.

"The least touch is acutely felt in rheumatism, whereas, to increase the pain by pressure in pleurisy, it must be made between the ribs, and pretty firmly. It is also to be distinguished by the partial or complete freedom from suffering when the ribs are fixed, and respiration is carried on by means of the diaphragm —by the circumstance of cough being absent, or at least not necessarily present, in rheumatism, whereas in pleurisy it is rarely wanting—by the degree of constitutional disturbance being much less than in pleurisy, if the rheumatism be confined to the parietes of the chest, while its appearance elsewhere, if it be not so confined, renders any other diagnostic mark almost superfluous." 19.

Rheumatism, however, is liable occasionally to attack any fibrous or tendinous structure in the body.

"The constitutional disturbance which attends acute rheumatism is of a well-marked and striking character. When any of the dense ligamentous structures are in a state of active inflammation, the general system sympathises largely with the suffering part; in fact, it would appear that the less vascular and more insensible the texture, the more ardent is the fever which is lighted up when it does become inflamed. So, in this form of rheumatism, we meet with nearly as violent specimens of re-action as we are ever called upon to witness. The chilliness or shivering with which this, in common with other acute fevers, is ushered in, having passed away, becomes speedily followed by great heat of skin, with copious but partial perspirations, which are almost invariably acid, rapidly reddening litmus paper, and often exceedingly sour to the smell. The pulse increases to 90, 100, or 110, in frequency, and has a peculiar character. In books it is usually stated, rather emphatically, not to be hard; but I do not think this quite correct. The pulse is large, full, and active: not so hard as the small, concentrated, incompressible, pulse of serous inflammation, but often quite as hard as a pulse of such size can well be supposed to become. The heat of skin and activity of the pulse bear a relation to each other, as might be expected; and sometimes both are less exalted than I have above supposed, the heat being moderate, and the pulse large, but soft. Such cases usually yield to treatment more speedily than the others.

The tongue, where the fever runs high, becomes deeply loaded, white, and clammy, or even yellowish and dry. No acute disease, except continued fever, exhibits so thick a fur, and the coating of the tongue is even more uniformly present in acute rheumatism than in the common fevers of this country. The appetite is impaired, but generally not so absolutely annihilated as in fevers proper; there is also much less urgency of thirst. The bowels are sluggish and loaded, the evacuations usually dark and offensive. The urine scanty, generally much, and sometimes prodigiously, loaded with the lithates.

A remarkable difference between this and other forms of fever approaching to it in violence, is the comparative infrequency of what are called nervous symptoms. Delirium is a very rare occurrence; indeed I have never seen it, so long as the rheumatic inflammation has been limited to the external parts, and in many very acute cases there is no headache from first to last." 22.

Under ordinary treatment, Dr. Macleod considers that this form of rheumatic fever lasts five or six weeks; but we know, from pretty extensive experience, that it "may be often speedily extinguished if met at the onset by appropriate means."

Where the heart has escaped injury, the recovery is generally complete; but the disease is very liable to relapse, when stimulants are taken too soon, or the patient is exposed to cold.

Our author makes many acute and practical remarks on the diagnosis between acute rheumatism and gout, which deserve the serious attention of the young practitioner.

### CHAP. III.—TREATMENT.

Very few diseases of such common occurrence have given rise to so much variety in the methodus medendi. Dr. M. thinks, and perhaps justly, that rheumatic inflammation has been too much separated, in treatment, from other phlegmasiæ.

"I presume, indeed, that most practitioners of the present day are in the habit of abstracting blood in cases of acute rheumatism; but so far as my observation extends, the depletion is adopted on principles very different from those which are acknowledged with regard to other inflammatory diseases. I mean, that the blood-letting is either looked upon as only favouring the operation of certain remedies which are to follow, or at all events as a means which may moderate, but cannot be expected to extinguish the disease; to accomplish which requires certain routine prescriptions not usually adopted in other inflammatory affections." 29.

In short, our author thinks that the *acute* form "is amenable to the same laws as other inflammations." That acute rheumatism requires all the auxiliaries of the antiphlogistic treatment, as far as regards rigid abstinence, open bowels, diluents, &c. we readily grant; but that it is to be treated like common inflammation—say pneumonia—as far as regards venesection, we demur, though with the greatest deference to our author. Thus in acute rheumatism you may bleed to the last drop in the body, and the buff and cup will remain.—Not so in other inflammations. It is extremely difficult to bleed to syncope in rheumatic fever; but we have seen this state produced several times; almost always with metastasis to an internal organ. Will this occur in pneumonia? Although therefore, the general principles of treatment in the phlogoses at large are applicable to the one under consideration, we hold that general bleeding, though necessary and proper in the early stage of the disease, and in unbroken constitutions, has not proved so useful, in our hands, when we have acute rheumatism to deal with, as when we have common inflammation, as of the lungs, kidneys, brain, or pleura. It may be very true, what our author says, that there is nothing *specific* in rheumatic inflammation, except what depends on the specific seat of the disease. This is all we contend for; because this demands a modification of treatment. We surely would not confound inflammation of the skin, in erysipelas, with acute rheumatism, whose seat is deeper, and so on, as far as the methodus medendi is concerned.

In answer to some practitioners who condemn blood-letting as injurious in this disease, Dr. M. relates the case of a young man who came into St. George's Hospital with "great tumefaction and redness of the left hand and wrist of only thirty-six hours' duration." Dr. M. was advised by a gentleman (whose father cured acute rheumatism very rapidly by copious venesection) to bleed to thirty ounces, and no medicine to be given. "In this case the disease was literally extinguished." All we say to this case is—"valeat quantum."

"To come more to particulars, I should say, that in well marked cases of rheumatic fever, within the first week of their onset, and in individuals of the average degree of robustness, from twelve to twenty ounces of blood may be abstracted with advantage, several successive times, in the course of five or six days. In judging of the propriety of repeating the venesection, the three points chiefly requiring to be attended to are, the state of the blood, the character of the pulse, and the effect produced upon the rheumatic pains.

The blood, as every one knows, is generally very much buffed in rheumatism, but the presence of this appearance is not sufficient to warrant, *per se*, the repetition of the depletion. I think, however, the converse holds good, and that a loose coagulum, without the buff, is a sufficient reason in rheumatism for avoiding venesection, even although the attack may otherwise be acute.

The pulse sometimes, though very rarely, loses its peculiar bounding character after the first abstraction of blood; and where this happens, I have not found further depletion of service.

As regards the pains, their cessation, or very great diminution, alone warrant us in declining to repeat the blood-letting; the general order of events being, that an abatement of suffering follows each recourse to the lancet. But whatever the aspect of the case might be, if no relief whatever resulted from bleeding practised once, or at most two several times, (an event which I have scarcely ever witnessed, except where some of the circumstances above-mentioned were present, and thus gave early notice of the peculiarity,) I should then substitute other remedies, and avoid pushing the depletion further." 33.

To this we see no particular objection. Dr. M. justly attaches great importance to purging. We have had such multiplied experience of the efficacy of this remedy that, were we restricted to any *one* measure, we should infinitely prefer the cathartic draught to the lancet. Both, however, may be advantageously employed. The purgative recommended by our author is from three to five grains of calomel at night, and the black draught in the morning. This he repeats on several successive nights and mornings—indeed, the aperient is to be employed throughout the whole course of the disease. In comparatively mild cases, this treatment, Dr. M. observes, will be sufficient.

Next to bleeding and purging, Dr. M. considers opium as useful in acute rheumatism. Two grains, on an average, in the twenty-four hours, he thinks sufficient. Dr. M. was one time in the habit of giving calomel with the opium; but does not do so, unless there is suspicion of some rheumatic inflammation invading the internal organs, when "mercury must be immediately administered and persevered in until such symptoms are entirely subdued." Dr. M. makes some judicious observations on "other expedients," as sudorifics, &c. He considers the mist. guaiaci as a useful remedy, especially in broken-down constitutions when bleeding could not be borne, and where the disease is subacute rather than acute. Colchicum, he thinks, is only useful in acute rheumatism, when it purges—and even then is not superior to many other purgatives. We are a good deal surprized at this observation; for in hundreds of cases—and personally ourselves—we have witnessed and felt the almost wonderful influence of colchicum in gout and acute rheumatism. We know, indeed, that it is always more beneficial when combined with aperients, and when it acts itself on the bowels; but still, if any medicine in the Pharmacopœia possesses a specific power—if ergot of rye acts on the uterus—colchicum exerts that specific influence on gout and rheumatism.

Dr. M. justly condemns cinchona; and how it could ever have been employed, puzzles us extremely. Even in convalescence we seldom dare prescribe it, as it often induces a relapse.

"I know no local remedies which afford any relief in acute rheumatism except the application of leeches. These, however, ought not to be had recourse to unless under particular circumstances; indeed the only cases in which I would recommend them are those wherein the pain remains very severe, and fixed in one, or perhaps two joints, after general depletions have already been carried as far as is deemed advisable. The suffering of the patient is generally relieved very much by leeching under such circumstances; but it is only to be regarded as a palliative, and in the great majority of cases is not called for." 40.

We have rather a better opinion of leeches than Dr. M. but they are by no means essential to the cure.

### Chap. IV.—Carditis and Pericarditis.

This is the most common complication of rheumatic fever. M. Bouillaud considers the *coincidence* of rheumatism and carditis as the rule, and the reverse the exception—and yet, in another place, he says that, in one half of the cases of acute rheumatism there was carditis! Dr. M. has too much good sense to adopt either of these opinions to their full extent; but still he evidently believes that not only a great proportion of acute rheumatic cases are attended with carditis, but that the idea of metastasis is quite illogical. The disease, he thinks, fastens on the heart, simultaneously with its invasion of the joints—or even before that sometimes.

" I have no hesitation, however, in expressing my conviction that the pericarditis supervening during rheumatic fever may more correctly be regarded as an extension of the disease, than as a true metastasis." 45.

Again—

" According to my experience, the heart affection is much more frequent in severe than mild cases of rheumatism. I do not mean to say that I have not seen pericarditis come on where the external rheumatism has been mild; but merely this—that such examples are rare, compared to those in which the converse holds good." 45.

Our own experience coincides with that of our author on this point. Dr. M. has not found pericarditis attend the other forms of acute rheumatism in anything like the proportion in which it does in this, the general or diffused kind. Dr. M. earnestly argues that blood-letting does not contribute to the occurrence of pericarditis, even when in large quantities and often repeated. We cannot say that his arguments have brought conviction to our minds—and certainly we are, of all people, the least prejudiced against venesection in general. Dr. M. does not allude to the subject of the warm bath in this form of the disease. It is a remedy too often employed, and, on numerous occasions, we have recorded our opinions in this Journal, that the measure in question was, of all others, the most conducive to an *extension* (for we dare not now say *metastasis*) of the disease from the joints to the central organ of the circulation.

### Chap. V.—Symptoms of Pericarditis.

These may be divided into two classes—the local and the general. Among the former, *pain* is the most common, though it is sometimes absent, even in the worst cases. When present, it is sometimes like an acute stitch, increased by inspiration, as in pleurisy:—but more generally it is a dull, heavy, burning uneasiness—or intermediate between these. Occasionally it is distinctly aggravated by pressure on the intercostal spaces. In first attacks the pain is usually circumscribed; but after repeated invasions, and where structural change has commenced in the organ, the pain is diffused over the left side.

"The extent to which the heart's action is altered, as indicated by the presence of palpitation, or of any peculiar affection of the pulse, is much less than we should *à priori* have anticipated, or than it is often represented to be, in books. Limiting, for the present, the term palpitation to that tumultuous action of the heart which directs the patient's attention to it, and induces him to make it the subject of complaint, I am quite satisfied that it is frequently entirely absent; while, with respect to the pulse, it not only has nothing which can be regarded as pathognomonic, but I should even say that it has no character which is calculated to afford us any material assistance in the diagnosis. Generally, indeed, it is increased in frequency (beating from 100 to 120,) and at the onset is also, I think, usually more jerking than natural; but this latter character often very speedily subsides, and then, although generally retaining its augmented frequency, it may become weak, unequal, or intermitting—all which phenomena, it is well known, may attend other diseases of the chest, more especially those attended with effusion." 53.

But auscultation by a practised ear is a very different thing. The abnormal sounds are chiefly two—rubbing or friction sound—and blowing or whizzing—the *former* appearing to result from the friction of the pericardium on the surface of the heart—the *latter*, on the impetus of blood through the valves. Effusion or accretion between the surfaces of the pericardium will put an end, of course, to the rubbing sound. Not so with the blowing sound.

"Where the blowing is heard with the first sound of the heart, and where it is perceived on placing the instrument over the carotid arteries, it is reasonable to conjecture that it depends upon disease having produced, in the aortic valves, certain changes which cause obstruction to the free exit of the blood from the ventricle. If the blowing be contemporaneous with the second sound, and not heard over the carotids, it probably will be found to depend on disease at the orifice between the left auricle and ventricle. Frequently a whizzing sound is heard with both actions of the heart, depending, in general, upon the aortic and mitral valves being both implicated in the disease. The degree to which the blowing sound is present varies much in different instances; but in well-marked rheumatism of the heart, it is rarely, if ever, wholly absent. Its general character, where the valves have not been previously damaged so as to admit of regurgitation, is that of a simple bellows sound; but sometimes it assumes a harsher character, and sometimes a distinctly musical tone. I have heard a clear musical whistle nearly from the onset of an attack of rheumatic carditis, diminishing in loudness as the disease was overcome, and passing into a simple bellows sound, which remained permanent. Indeed, there can be no doubt but that this anormal sound may entirely cease in recent attacks, the causes on which it depends being, it is to be presumed, wholly removed; but in older cases, and especially in those where the heart affection has not been early and vigorously met, the sounds alluded to are permanently present, affording every variety of intensity and tone, and too often resisting every method hitherto attempted for their removal—a circumstance obviously depending upon irremedial structural changes having been produced. And this leads me to remark, that it is of great importance, in cases of acute rheumatism, to ascertain, at the earliest possible period, the condition of the heart, that we may not confound the phenomena produced by the attack then present with those resulting from previous organic change." 57.

Sometimes, though not generally, the cardiac region is dull on percussion, to a considerable extent, and which, in the healthy condition of the organ, is limited to a couple of square inches, or less. When this dulness comes on during acute rheumatism of the heart, we may set it down to the account of effusion into the pericardium—if, after repeated attacks, hypertrophy of the organ will be the probable cause of the dulness.

"Of the general symptoms which indicate that the heart has become implicated in an attack of acute rheumatism, the most striking is the aspect of the patient. I venture to say there is no observant practitioner who has not had occasion, on going into the wards of an hospital, to stop at once on coming to a rheumatic patient whom he may have seen the day before apparently doing well, and proceed to examine the heart with the conviction on his mind, before he has asked a single question, or applied his stethoscope, that carditis has supervened in the interval. This is one of the many instances in which the eye can detect what the pen cannot express. The system has taken the alarm at the new inroad of the malady; the consequent distress is depicted in the countenance, and told in every attitude and every movement. The expression is anxious; the breathing rather shallow; occasionally there is cough. The patient is sometimes very restless, but more generally lies on the back, or right side; at least it is rare to see him choose the left. Here we have intense fever, but for the most part without the restlessness and tossing that usually attend that state. Indeed there is occasionally a fixedness in the general aspect—I had almost said in the deportment of the patient—quite remarkable; he becomes, as it were, passive, and while the immobility with which he retains one position would lead us to suppose that any other would be intolerable to him, yet I have known such a patient, upon being moved, remain in his new position apparently as determinedly as he had previously done in the other. Although, therefore, reclining on the back, or a little to the right be the most common, and therefore, we must presume, generally the easiest posture, yet the unwillingness to move, even to resume this attitude, probably depends upon the effect produced by motion of any kind on the heart's action, which thus becomes for the time still further embarrassed. In the cases where this unwillingness to any change of posture is most marked, the action of the heart is usually feeble, and the sounds indistinct. Dr. Latham, who has excellently described this phenomenon, attributes it, and probably with justice, to the presence of fluid effusion in the pericardium. And here I may observe that where copious effusion takes place rather rapidly into the pericardium, unaccompanied by hydrothorax, the patient will sometimes prefer to lie perfectly flat on the back, without having the shoulders raised at all; being just the reverse of what we are almost invariably taught in books. Such, at least, has been the fact in several cases in which I had an opportunity of examining the bodies, and in others where the patients recovered, after having had symptoms which led me to believe that there had been fluid in the pericardium." 61.

A state of the encephalon sometimes supervenes on pericarditis resembling delirium—or wild insanity. It is generally a fatal symptom, and yet the traces of inflammation in the brain or membranes, is rarely discoverable after death. Dr. M. has, however, met with a few cases where recovery took place.

Chap. VI.—This chapter is on the immediate effects of inflammation (rheumatic) on the pericardium and heart. When the treatment is energetic, rheumatic pericarditis is generally cured, in primary attacks; but otherwise the organ receives an injury which leads to future change of structure that is irremediable—too often fatal. Some, however, are cut off in the first attack, and the appearances on dissection are then unequivocal.

"The results to which the inflammation has ulteriorly given rise, are sometimes in description confounded with those which spring directly and immediately from this cause. In a recent case the appearances are striking. On raising the sternum, lymph is sometimes perceived even in the anterior mediastinum. The bag of the pericardium is seen to be inflamed; and here, unlike what I had occasion to mention with respect to rheumatism of the joints, the effects are conspicuous, and the inflammation marked by a greatly increased number of vessels carrying red blood. The pericardium feels pulpy, or fluctuating; and frequently on cutting through it we do not at once expose the heart, but find a layer of lymph intervening, adherent partially or more extensively to both serous surfaces.

But the anormal appearances are generally not limited to the exterior. The valves, especially those on the left side, may be inflamed. Ulteriorly the change is manifested by their augmented size and thickness; but at a very early period, and supposing it to be a first attack, the only thing which may be visible is what might be described as a slight tumefaction at the ostea: looking from the left auricle towards the ventricle, the aperture seems narrowed: and I have seen the portion of the valve where its sides meet in closing, of a flesh-colour, presenting through a magnifying glass the appearance of minute spots, like granulations, with vessels carrying red blood entering them from the interior of the auricle. The aortic valves are sometimes of a pink hue to the naked eye, though there may be no separate red vessels to be detected even with a glass. Points like very minute granulations may sometimes be perceived, being apparently the earliest stage of those small bead-like projections which are frequently seen at a more advanced period. M. Chomel has also described the appearance of granulations, varying in size from a pin's head to a millet-seed, as occurring in a recent case both on the mitral and aortic valves; the former affording unequivocal evidence of being inflamed by having upon it a portion of adherent lymph. In the case to which I have above alluded, there was perhaps some narrowing of the aortic orifice, as if the parts around had been slightly tumefied; but this was not so conspicuous as in the auriculo-ventricular aperture. Red vessels could be traced from the surface of the auricle to its valve, but not so with respect to the ventricle. On the right side there was some opacity of the mitral valve, but nothing further. The pulmonary valves were healthy. This is the earliest condition in which I have had an opportunity of examining the endocarditis which accompanies rheumatism—that morbid change in the valves of the heart which ultimately leads to their permanent opacity and thickening, and to various important alterations, not only in the heart, but also as a secondary consequence in other parts of the body." 70.

In our post-mortem examinations, however, we are more frequently called on to witness ulterior than early consequences of rheumatic carditis. These are thickening and opacity of the pericardium, especially of that part which is reflected over the heart—adhesion of the two surfaces, general or partial—adhesion of the external pericardium to neighbouring parts, &c. But these changes, though important in themselves, are trifling, compared with the internal disorganizations. The valves, from their pre-eminently fibrous and tendinous structure, are the greatest sufferers in rheumatic carditis—especially the auriculo-ventricular valves—mitral and tricuspid.

"By much the most common kind of change is more or less of thickening, and this, more especially in the mitral valve, is sometimes accompanied by unequivocal evidence of previous inflammation of the adhesive character, its different portions being frequently more or less united together, or glued to the adjacent surface of the heart. When the auriculo-ventricular valves on either side are opaque and thickened, the participation of the lining membrane beyond them in such change is for the most part much more marked in the auricle than in the ventricle; corresponding to what I have already stated with regard to the acute and earlier stage of the disease." 77.

It is very rare to find the muscular structure of the heart itself inflamed in rheumatic pericarditis or endocarditis—our author never having seen an unequivocal example of the kind. The parietes of the organ, however, undergo changes which may be considered mechanical in their causes—as hypertrophy, with or without dilatation of the chambers—or attenuation of the parietes with augmentation of capacity in the ventricles or auricles. These changes are generally attributed to the obstruction which the heart finds in distributing the blood to the aortic and respiratory system, and consequently to the greater efforts which it is called upon to make. We suspect, however, and so does Dr. M., that there are other causes, besides the mechanical, which operate in producing these morbid changes.

"The most conspicuous symptom, supposing the nervous energy of the patient to be unimpaired, is to be found in the action of the heart. This is more turbulent than natural; the contraction of the ventricle occupies a perceptibly increased period, is accompanied by a kind of heaving, and there is frequently a greater than natural projection of the chest at each pulsation, which is very characteristic. If one end of the stethoscope be placed over the heart, and the fingers applied to the other extremity, the blow may not only be felt but seen, by the impetus communicated to the instrument. There is also a peculiar prolonged swell in the arterial pulsations. If there be great enlargement with thickening, then, in addition to the above sign, there is also dulness on percussion over a preternatural extent of surface, corresponding to the increased size of the viscus. If there be dilatation without hypertrophy, the dulness generally exists over an increased space, but without the augmented impulse; and these very simple diagnostic marks are sufficiently clear to afford the means of forming an opinion which will be correct in the great majority of cases. It is remarkable how frequently patients labouring under hypertrophy and dilatation, with the increased force and duration of the heart's action to which they give rise, are yet themselves unaware of anything unusual. I have repeatedly questioned such persons, and have often found that, unless under excitement, they felt nothing unusual about the heart; and I can venture to say, that, as a general rule, they do not complain nearly so much as those labouring under merely nervous palpitations without any organic change.

Another almost constant attendant of an old rheumatic heart is the blowing sound; and, as this generally bears a relation to the extent of valvular disease and to the force of the heart's action, it is reasonable to conclude that it depends upon the passage of the blood through the ostea of that viscus, whether in the proper course of the fluid, or by regurgitation.

In the great majority of cases, the phenomena presented are increased size and thickness of the valve; but notwithstanding its increased dimensions, it very generally does not close the auriculo-ventricular aperture, which, if the disease be of long standing, is yet more considerably augmented. It is to be kept in mind, however, that the blowing sound may also arise from causes apparently independent of any permanent disease.

This sound also varies much in its intensity and tone, passing by insensible gradations from a soft blowing to a harsh whizzing or rasping sound, and in some—not *very* rare instances—even striking a distinct musical note; perhaps more like cooing than any thing else with which it can be easily compared. This was the simile used by Dr. Elliotson, who, I believe, first observed and described the phenomenon, and I know no better mode of conveying a correct idea of it." 84.

### CHAP. VII.—TREATMENT OF PERICARDITIS.

Dr. M. only adopts a modification of the treatment employed where the heart is not implicated. "The chief remedies in rheumatic inflammation of the heart will be found in bleeding and mercury." The venesection here requires more caution than when the joints only are affected. "For, while its necessity is augmented, the power of sustaining it has been diminished by the function of the part now implicated in the disease." Smaller and more frequent bleedings than in rheumatic fever are here recommended, in order to avoid syncope. Fourteen ounces are regarded as an average quantity, twice, or even thrice in the course of the first twenty-four hours. Local depletion is here of more value than in the articular forms of rheumatic inflammation.

"Lymph effused about a joint may render it stiff and troublesome, but when effused on the surfaces of the heart, it may be destructive of the function of the organ, and with this of the life of the individual. We must therefore direct our treatment more energetically, and for a longer period, against this effect of the inflammation. I allude to the use of mercury, which must be administered so as to bring the patient as speedily as possible under its influence, and the effect of the remedy must be kept up till we are satisfied that it has done all it is capable of effecting towards the removal of the lymph which had been poured out. Of the different preparations of mercury for this purpose, calomel is, I believe, beyond all comparison the best, and a scruple in twenty-four hours may be stated as forming the average quantity which adults under such circumstances require at the onset of the attack; the quantity being immediately diminished, but the remedy not abruptly discontinued, on the gums becoming tender. It is always to be kept in mind, too, that the object is not to make the mouth sore, but that the soreness of the mouth is merely to be looked upon as evidence of another and more important object having been accomplished—namely, bringing the system under the influence of this most powerful agent" 87.

Purging freely, at the commencement of pericarditis, is necessary; but after effusion the laxatives must be moderate, in order not to check the mercurialization, "which then becomes paramount." Blisters to the region of the heart, when symptoms of effusion are shewing themselves, become useful.

"The objects in view are to extinguish the inflammation at its onset if possible, before lymph has been poured out, for which purpose bleeding and purging are the best expedients; but if we fail in this, as in acute cases we generally shall, unless we have an opportunity of treating them at the very onset, then the mercurial action, vesication, and moderately open bowels, are the remedies to be employed." 89.

Quietude of body, and, if possible, of mind, after these attacks, is obviously useful, as morbid irritability of the organ remains for many months.

### CHAP. VIII.—CAPSULAR RHEUMATISM.

If difference of seat, symptoms, termination, treatment, &c. be sufficient to prove a distinction, then synovial rheumatism ought, Dr. M. observes, to be dissociated from the form which we have been considering.

"The seat of capsular rheumatism is in the lining membrane of the joints and bursæ of the tendons; being, in this respect, the same as gout, to which it is, in various respects, very closely allied. The parts most liable to its attacks are the feet and hands, where it is, for the most part, easily recognised by the enlargement of the joints; but the peculiar characters of the disease are, perhaps, most strikingly displayed when it attacks the knee. At first there is pain and heat, which, in the more severe cases, are attended by external redness; and after a time, varying from about twenty-four hours to several days, tumefaction of the parts takes place. The appearance of the swelling, which is limited to the immediate vicinity of the joint, points out the nature of the affection. The effusion is within the synovial capsule, which is consequently distended, and projects at those points where it meets with least resistance. This gives a peculiarity to the shape which at once strikes the eye. In the knee, the fulness is most conspicuous on either side of the patella and across the lower part of the thigh, just above the joint: frequently also it projects backwards, and may be seen or felt between the hamstrings. Even where the inflammation extends to the surrounding textures, they participate much less in this than in the preceding form of the disease, and the swelling is consequently much less diffused. In the synovial rheumatism the swelling is fluctuating, and bound down by the ligaments of the joint; in the common acute rheumatism, the effusion is external to the capsular ligament—chiefly in the cellular membrane —and does not fluctuate. The shape and aspect of the two thus differ strikingly from each other, and only require to be seen in order to be afterwards recognised without the least difficulty, unless there be something very unusual in the case." 93.

The capsular rheumatism is much more persistent, and, though affecting several joints, at first, becomes fixed in a limited number, often leading to permanent change of structure in the part.

"In general, it is probable that the effusion into the joint consists merely of an increased quantity of synovia, produced by the excited condition of the secreting surface. Such, at least, was the case in an instance wherein I had an opportunity of examining the body of a patient cut off by another disease, while labouring under a first attack of synovial rheumatism. The joint chiefly affected was the knee; it contained an increased quantity of synovia, and the lining membrane, except where reflected over the cartilages, was of rather a deep red colour, and tumefied. But where the attacks have been repeated, or have expended themselves upon particular joints, much more considerable changes take place." 94.

Each repetition of attack is followed by less perfect recovery, and more distortion and disfiguration of the joint, with loss of motion, but not much pain.

Dr. M. dedicates some pages to prove that synovial rheumatism sometimes terminates in suppuration, and adduces cases as well as authorities in support of this position.

"The first indication of this form of rheumatism generally consists in a dull aching and stiffness of certain joints, particularly those which are most superficial. After a time the pain becomes of a more acute or burning character, and the parts begin to swell, the enlargement presenting a model of the synovial or bursal cavity, into which the effusion has taken place: the suffering is increased either by stretching the parts much, or by corrugating them, and hence a semiflexed position of the joint is the one usually assumed in severe cases. Motion aggravates the pain, particularly after rest of some continuance, as in the morning, at which time there is often a great deal of stiffness about the parts; but in chronic cases this subsides after a little exercise, and the limb assumes more of its wonted suppleness.

In the most acute cases there is smart fever, and some redness about the joints; but much more usually there is no external discoloration, and little fever; at least the fever is inconsiderable as compared to that which attends the form of rheumatism first described. When fever is present to a considerable extent, it generally presents the remittent type, with copious perspirations; and I have seen a pulverulent deposit on the skin, which admitted of being scraped off, in quantity minute indeed, but sufficient to admit of being tested, and which was found to consist of the lithate of soda. Where the fever runs rather high the tongue becomes loaded and the bowels constipated; but there is much less disposition to these states than in common rheumatic fever. Indeed, of the secretions, the perspiration and urine are those most uniformly affected, the latter being charged with the lithates, and sometimes producing a sense of scalding as it is voided. In one case, which lately occurred to me, this was attended with slight discharge from the urethra, although I have the strongest reason to be assured that there was no gonorrhœal affection. The phenomenon would seem to bring still more close the connexion between the urethra and synovial membranes.

The course of this disease is altogether considerably different from that of rheumatic fever; it more speedily becomes mitigated in severity when both are treated in the ordinary manner, or when both are left to the unaided efforts of nature. But it is rarely if ever cut short at once, and the subacute, or chronic condition, is much more enduring. In a first attack, from three to six weeks may be stated to be the average duration of the illness, and in such case little or perhaps no perceptible damage may have been done to the affected joints. But it is very apt to recur, and with each relapse its obstinacy seems to increase; in fact, the inflammation acting repeatedly on textures already diseased, at length may be said never to be wholly absent, giving rise to permanent swelling and stiffness about the joints, constituting the 'nodosities' already described. Sometimes, after years of suffering, a truce is at length entered into, and the patient enjoys a long respite, without our art having any apparent share in the favourable change." 111.

The attack on the superficial joints is easily recognized; but in the deeper-seated, as the hip, there is more difficulty. In such cases, however, there is generally some external part implicated, which gives us a clue to the mystery. When a deep-seated bursa only is affected, we have only the exciting causes and the constitution of the patient to guide us.

Chap IX.—In this, the capsular form of rheumatism, pericarditis is very rare. In five cases of synovial rheumatism, wherein patients have died from transference of the inflammation from the joints to internal parts, no affection of the heart was found after death,

"Indeed, there can be no doubt that, on the great scale, there is a striking difference between the affections of internal organs in rheumatic fever, and in cases of capsular rheumatism, even in its acutest varieties. While the former implicates both the external and internal membranes of the heart by *extension*, and this in a very large number of cases, the latter (where it involves an internal part,) becomes transferred to the pleura, or to the membranes of the brain, by *metastasis*, and this in but a small proportion of instances. Nor are the immediate results of such internal inflammations less remarkable; for while very few, if properly treated, die during the first onset of rheumatism of the heart, the mortality holds a very high ratio where the synovial inflammation becomes transferred to the serous membranes either of the head or chest." 114.

It is in this form of rheumatism that our author has found the head to suffer much more than in the form previously described:—and this, not when the disease has been erratic, but where it has been fixed in one or more joints for some time.

"Sometimes the affection of the brain is manifested by pain in the head, which is at one time acute, at another dull—at one time persistent, and at another periodic, or at least paroxysmal, with almost complete remissions. Where there is pain in the head, however slight, attention is generally directed to the part; but occasionally there is no pain whatever, even although the disease has made considerable advances.

The following case may serve to illustrate the peculiarities of one form of this disease:—

A gentleman of literary habits, in the 37th year of his age, had suffered for above two months from pain, with swelling and fluctuation, of several joints, particularly the knees. The right knee was more especially complained of, and there was considerable effusion into the joint, evinced by a soft fluctuating projection on either side of the patella and across the lower part of the thigh, just above the knee. He took each night acetic extract of colchicum, in doses of gr. iij. and acetate of morphia, which last was increased gradually from $\frac{1}{4}$ to $\frac{1}{2}$ a grain; but without any effect in relieving the affected part. About the end of May, 1837, he complained of his memory being impaired, so that he had great difficulty in remembering words; but he was able to go out in an open carriage, and took a short airing, on Sunday, June 2d.

Next day I found him in bed, unable to answer questions otherwise than by the monosyllables 'Yes,' and 'No.' He expressed his mortification at this inability to speak, by gestures, and when asked if he had any pain or giddiness about the head, answered '*No*;' and shook his head in such manner as to show that he perfectly understood what was said to him. His pulse was 78, and soft; his tongue clean. No complaint was made of the knee, but on examining it I found the swelling almost entirely gone; indeed all fluctuation had disappeared, and there remained only some puffiness about the parts. He was freely purged, and a mustard poultice applied to the knee.

4th.—The same symptoms continued without perceptible change. Twelve leeches were applied to the temples, and the purging repeated.

5th.—In the early part of the day he seemed better; recollecting his sister's name, which he had previously forgotten. Late at night was seized with a fit of screaming, accompanied by strabismus affecting the right eye, and followed by frequent moaning.

> Leeches to forehead, and gr. ij. of calomel every three hours. A blister to the back of the neck.

6th.—Strabismus gone, but mouth perceptibly, though slightly drawn to right side, with much subsultus tendinum, and frequent sighing. Answers questions which require only monosyllables distinctly; says '*No*,' when he is asked if he has pain in the head, and '*Yes*,' when asked if he has any giddiness. Pulse 80, with some sharpness.

> Twelve leeches to temples; calomel, gr. iij. every three hours; strong mercurial ointment to be rubbed in very freely.

7th.—Very little change. Calomel omitted after the seventh dose, in consequence of purging. Mercurial frictions continued every two hours.

8th.—Much more sensible, and expression improved. Urine, which has been rather scanty, has become more abundant. Pulse 78. No mercurial fætor, but a perceptible red line on the gums.

9th.—Continues apparently to improve; gums decidedly injected, but there is no mercurial fætor. Takes beef-tea plentifully. Shows by his manner that he recognizes those about him, but cannot name even his most familiar friends.

Towards evening he began to sink. Frequent starting, and constant deep sighing; pulse became diminished in power, while it rose in frequency to 100. These symptoms continued till the evening of the 10th, when he expired.

*Autopsy.*—The convolutions of the brain were flatter than usual; the arachnoid was injected and there was a slight appearance of effusion. The ventricles contained from oz. iss. to oz. ij. of clear limpid serum, and each corpus striatum had the appearance of being smeared over with a thin layer like cream; but this could not be wiped off, and apparently depended on thickening and opacity of the lining membrane. The convolutions of the left fissura Sylvii were adherent, from inflammation of the interposed membrane, and the substance of the brain on either side of the fissure was of a yellowish colour, with patches like minute points of extravasation. The heart and other thoracic viscera were perfectly healthy. In the right knee, which had been the chief seat of the rheumatism, the membrane was thickened, rugose, and red all round, close up to the cartilages. The membrane had been pushed upwards, so as to increase the extent of the cavity, but no longer contained any preternatural quantity of synovia.*" 119.

Our author has seen several analogous cases where slow and treacherous inflammation of the brain has crept on, without much alarm, to a fatal termination. A case occurred in St. George's Hospital, under Dr. Seymour, where a sudden collapse took place in joints that had been long distended, succeeded by pain in the head, hemiplegia, and death in the space of 36 hours. On dissection there was found considerable effusion into the ventricles, with a deposit of greenish-looking purulent matter over the surface of the left hemisphere.

"Another complication of this form of rheumatism is with pleurisy; and here, too, the internal inflammation depends upon a metastasis, the affection of the joints subsiding, or wholly ceasing, on the supervention of the thoracic disease. I have more than once witnessed this occurrence, and the phenomena were rather remarkable, having given rise to a copious sero-purulent effusion into the bag of the pleura. This form of metastasis is quite distinct from that which takes place to the pericardium in diffuse rheumatism. In this last the inflammation may, and frequently does, extend over the covering of the heart to the contiguous pleura: but in the cases of metastasis of synovial rheumatism to which I allude, the pericardium was free from any participation in the disease." 121.

In respect to rheumatic ophthalmia, our author has observed but few cases of it, though the term is very liberally employed by ophthalmic writers, with no inconsiderable vagueness.

CHAP. X.—This Chapter is on the difference of treatment in diffuse and capsular rheumatism. The difference is very considerable in Dr. Macleod's estimation. General blood-letting, so useful in urgent cases of the *former*, is here "rarely if ever required, while local depletion is often of the most essential service." Leeches can always be applied in articular rheumatism, and the relief they afford is of the most marked description. Evaporating lotions tepid are very soothing in general, and, while soothing, ought to be continued. In some, anodyne fomentations are beneficial.

---

* "This case was attended by Dr. Pereira, Dr. Clendinning, Dr. Hope, and myself."

"Internally, colchicum is the great remedy; and there are various modes in which it may be given. In the acute stage, the plan I have found to answer best is to give about a drachm of the vinum colchici in divided doses in the course of the twenty-four hours, combining the portion taken in the morning with a saline purgative draught, such as the common black dose. At the onset it is desirable to act pretty freely, but not violently, on the patient's bowels; and where the senna and salts prove too active, a drachm of the sulphate of magnesia, and thirty minims of the colchicum wine, may be given, in any light aromatic infusion. Where the bowels are very irritable, as sometimes happens in this form of the disease, a scruple of magnesia may be substituted for the Epsom salts. Another form which frequently answers extremely well, is to prescribe a drachm of colchicum wine and half a drachm of magnesia in a mixture, of which the third part is to be taken three times a day." 127.

With some trifling modification (some calomel, opium and extr. colchicum at night) the above is the plan, when rendered a little more purgative, which we have used, for more than a quarter of a century, in the common or diffuse form of rheumatic inflammation. We believe we have been as successful as our neighbours, for there is hardly a complaint in Cullen's Nosology which we are less afraid of than rheumatic fever, when early encountered. Dr. M. has not found opium so useful in this as in the diffuse form. When colchicum acts, as it often does, as an anodyne, opium is unnecessary; if not, the opium is to be had recourse to.

In a great majority of cases, the above treatment will materially mitigate the severity of the symptoms; but this form of rheumatism is very prone to lapse into the chronic state, and then induce organic changes in the parts affected. In this stage Dr. M. recommends from one to three grains of the acet. extr. colchici at night, with a quarter of a grain of acetate of morphia, or five grains of Dover's powder. Moderate purging is necessary when anodynes are given. We quite agree with Dr. M. in the following sentiments.

"There can be no doubt that the colchicum is an agent of great power, and such as to require some caution in its administration; but it has always appeared to me that the presence of the disease in question has a counteracting influence, preventing the injurious consequences which might otherwise ensue; just as we see in many cases that the energies of the most powerful remedies are held under control by the state of the system—a familiar illustration of which is afforded by tetanus in respect to opium. However this may be, I have never seen any injury whatever done by colchicum in the doses above mentioned, unless persevered in under the following circumstances—1st, The cessation of the pain; 2d, a depressed state of the nervous system; 3d, the occurrence of purging; 4th, the disappearance of the lithates from the urine. This last, I believe, was first pointed out by Dr. Graves, and is an indication which it is of importance to keep in mind.

The pain, according to my experience, is generally relieved by colchicum, independently of its purging; but the bowels must be excited to a moderate extent by other medicines, to prevent accumulation, or if they act from the colchicum with any violence, the remedy must forthwith be discontinued." 128.

Dr. M. is not a friend to the exhibition of calomel in this form of the disease, except in combination with purgatives, where the biliary secretion is vitiated.

When no farther improvement takes place from the colchicum, the iodide of potassium is often highly beneficial, especially where thickening has been left about the joints. Two grains thrice a day, Dr. M. considers a sufficient dose of this powerful medicine. We never exceed this quantity, and often fall short of this dose. We every day see it prescribed in doses by far too large.

"When the disease has become entirely chronic, it is of great importance to attend to the general health, and light bitters, with a little potass or soda, are frequently of essential service; nay, in some cases of this kind, where the debility is considerable, even the decoction of cinchona may be given with great advantage.*

Some assistance, but certainly not so much as could be desired, may be derived from local applications in the chronic form of the disease; one which I would particularly recommend is, pouring tepid or hot water upon the part. This combines fomentation with a certain degree of friction, and the temperature of the water, as well as the height from which it is allowed to fall, must be regulated by the feelings of the patient. Liniments and embrocations are generally useless, and if incautiously used are frequently injurious. When the thickening is quite in a chronic state, the ointment of hydriodate of potass very gently rubbed upon the parts, or painting them with tincture of iodine by means of a hair pencil, so as to produce desquamation, is sometimes of service." 131.

Affections of the brain consequent on articular rheumatism can only be treated on general principles, and as affections arising from other causes. The most imminent danger is that of effusion, and the other results of inflammation in general. Depletion and mercurial action are the best preservatives against consequences.

### CHAP. XI.—MUSCULAR RHEUMATISM.

This is a chronic affection, and different, in our author's estimation, from the sequelæ of acute rheumatism. Its most common seat is the muscles moving some of the large joints, as the hip or shoulder. The loins, too, are often affected, and sometimes the muscles of the neck.

"This form of the disease consists, in the milder cases, of a dull uneasiness in the part while at rest, which, however, becomes converted into an acute cutting pain when it is moved. Sometimes, indeed frequently, there is no discomfort except on calling certain muscles into action, and if this, from forgetfulness or other cause, be done abruptly, the suffering is often so acute as to make the patient cry out involuntarily.

Lumbago is a very characteristic form of muscular rheumatism. It occupies the loins, and is often aggravated to torture by any unguarded movement implicating the muscles of the part; but if the patient remain perfectly quiet, he is comparatively free from suffering. When very severe, he may be obliged to remain in bed, and very often is confined to the sofa. Even when he is able to walk about, he often does so in a semibent position, being unable to raise his body into a completely erect posture for some time after he has risen; nay, in some cases he cannot straighten himself at all.

Pain of exactly the same character not unfrequently attacks the muscles of one side of the neck, and the head is generally held awry to relieve the suffering

---

* "I think that in the case above supposed, and in almost all others where debility is the urgent symptom, that the cinchona is to be preferred to quina."

part. Either of these may be brought on by sitting in a draught, or otherwise exposing the parts to cold, especially if combined with moisture.

Another very frequent seat of muscular rheumatism is the intercostal space, where it is difficult to say whether the muscular or fibrous tissues are involved; and indeed the pain on inspiration is often as acute as the stitch of pleurisy. From this, however, it is easily distinguished by the absence of pain, and the complete relief afforded by fixing the ribs, and breathing with the abdominal muscles.

I have also more than once seen the same kind of affection in the abdominal muscles, the pain being completely controlled by putting a roller tightly round the body, or otherwise preventing the muscles from acting." 135.

In this, as indeed in all forms of rheumatism, the pain is much greater in the night than in the day. The obstinacy of chronic muscular rheumatism is proverbial, and is much aggravated by atmospherical vicissitudes. It often occasions, when long continued, a wasting of the limb.

" Purgatives are very frequently of great service in the different forms of muscular rheumatism, more particularly in lumbago, and as a general rule ought to precede the use of other remedies. The best plan, where there is nothing to contra-indicate its adoption, is to give from three to four grains of calomel at night, followed by a black dose next morning, and to repeat this once or twice during the first week; after which it is sufficient to regulate the action of the bowels, and to give rather a brisk purgative about once a week.

But in the kind of rheumatism to which I now refer, where it has not been preceded by the acute stage, and where the muscles are chiefly affected, remedies of the warm or stimulating kind are decidedly most useful. Among these, none has appeared to me to be so frequently efficacious as the ammoniated tincture of Guaiacum. Half a drachm is a sufficient dose with which to begin; but most persons bear—and indeed require—a drachm, three times a day, and with some it is necessary to increase the dose to two drachms or more. The most common sensible effect is that of purging the bowels, and where this is the case to any great extent the dose must be either diminished or discontinued. In most patients, however, it may be so managed as to regulate the alvine evacuations; and the tendency to diarrhœa may be prevented without inconvenience, by means of opium, which, in doses of about a grain each night, may assist materially in the curative process. There are two other forms in which this remedy has been frequently used in rheumatism, namely, the mistura guaiaci, and the compound familiarly called the 'Chelsea pensioner.' The first I think less efficient in this form of the disease than the ammoniated tincture; the latter (composed of guaiac, rhubarb, cream of tartar, sulphur, and nutmeg,) may be substituted with advantage for the tincture in those cases which prove very obstinate and lingering. The general effects which the remedy produces in any form which agrees with the patient, and which is likely to prove serviceable, are an agreeable sense of warmth in the stomach, followed by moderate perspiration, or, where the surface is kept cool, by increased action of the kidneys. In full doses it sometimes produces an eruption on the skin like urticaria; but I have never seen any serious inconvenience result from this." 139.

We shall not allude to the numerous external agents, as oils, balsams, mustard, horse-raddish, turpentine, &c. not forgetting the cod-liver and Cajeput oils. We were glad to see Dr. M. as if by an after-thought, glance at the warm bath in acute and sub-acute rheumatism.

" In the acute forms of rheumatism, which we have previously considered, probably no one would think of having recourse to the warm bath, in their early stage, nor has any benefit resulted from this remedy in any of the cases of the sub-acute form in which I have seen it used." 140.

We can assure Dr. M. that scarcely a day passes in which we do not see the effects or learn the history of warm-baths in rheumatic fever. Had it not been for this, we should not, for so many years past, have been in the habit of warning our junior brethren against this injurious practice.

The local application of vapour and the douche is very beneficial in chronic rheumatism, especially where there are nodosities and other sequelæ of the different forms of the disease. Acupuncture and electricity are not held in much repute by Dr. M.

### CHAP. XII.—WHAT IS NEURALGIC RHEUMATISM?

"I would apply the term neuralgic rheumatism to certain painful affections following the course of various nerves, and brought on by exposure to cold. The identity of these, however, with genuine rheumatism is more doubtful than with regard to any of the cases previously described. But I have so frequently seen this form follow the same causes, yield to the same remedies, and alternate with attacks more particularly of the arthritic rheumatism, that I cannot but regard them as of the same family." 142.

In this sentiment we entirely agree with our author. Sciatica is a familiar example, and the facial neuralgia, commonly called TIC, often alternates with sciatica and neuralgia of other parts. When common means fail, which they too often do, Dr. M. has had recourse to stramonium, veratria, aconite, and other heroics, with partial success. From the tincture of aconite (five minims thrice a day) he has seen more benefit in rebellious cases than from other poisons. Belladonna externally is by no means to be despised as an auxiliary.

### CHAP. XIII.—PERIOSTEAL RHEUMATISM.

Dr. M. justly observes that, in almost all cases of nodes, practitioners are too apt to set down the cause as syphilitic or mercurial. He has seen "numerous instances" where neither the one nor the other cause could be suspected.

"The treatment of this form of rheumatism is generally by no means difficult, though sometimes tedious. The acute cases, indeed, yield speedily enough when actively dealt with; but in the chronic form it is otherwise. In the former, leeches applied locally, with calomel and opium given internally, and followed by rather brisk purging, are sufficient under all ordinary circumstances to arrest the disease.

When, however, the case has passed into the chronic state, the iodide of potassium, in doses of from two to four grains, twice or three times a day, very seldom fails to give speedy relief, and is generally sufficient to bring the case to a favourable termination without the assistance of any other remedy. Where it falls short of this, the cure may often be completed by sarsaparilla, and sometimes the two remedies in question require to be alternated. Very often the iodide removes the swelling and pain in the course of a few days; but where it does not, then this latter symptom must be controlled by means of opium. Purging is not necessary in the chronic form of the disease, but the bowels, if disposed to constipation, require the assistance of gentle laxatives.

Blisters over the thickened periosteum are frequently of considerable service; and in obstinate cases farther relief is sometimes obtained by painting the parts with tincture of iodine, in the manner already described.

In this form of the disease, accompanied as it so often is with a cachectic state of the general system, the warm bath is occasionally of considerable service, and is generally very grateful to the patient." 150.

The work terminates by tables and references drawn from 387 cases of fibrous, capsular, and muscular rheumatism; and from 52 cases of rheumatic pericarditis. These tables are curious and useful, and are well worthy of attention. We have analyzed the work before us so carefully and largely, that we have enabled our readers to appreciate well the value of the publication. The pains, indeed, which we have taken to diffuse its important contents throughout the profession, on both sides of the Atlantic, are the best proofs of the kind of estimation in which we hold this small, but highly useful and practical treatise.

